# Immunotherapy: Review of the Existing Evidence and Challenges in Breast Cancer

**DOI:** 10.3390/cancers15030563

**Published:** 2023-01-17

**Authors:** Yun Hu, Yan Li, Zhangcheng Yao, Fenglin Huang, Hongzhou Cai, Hanyuan Liu, Xiaoyi Zhang, Junying Zhang

**Affiliations:** 1 Department of Breast Cancer, Jiangsu Cancer Hospital, Jiangsu Institute of Cancer Research, The Affiliated Cancer Hospital of Nanjing Medical University, Nanjing 210009, China; 2 Department of Radiology, The Fourth School of Clinical Medicine, Nanjing Medical University, Nanjing 210029, China; 3 Department of General Surgery, Nanjing First Hospital, Nanjing Medical University, Nanjing 210012, China; 4 Department of Radiology, Jiangsu Cancer Hospital, Jiangsu Institute of Cancer Research, The Affiliated Cancer Hospital of Nanjing Medical University, Nanjing 210009, China

**Keywords:** breast cancer, immunotherapy, monoclonal antibodies, PD-1/PD-L1

## Abstract

**Simple Summary:**

Breast cancer is one of the most common malignant tumors in women. Its incidence rate accounts for 7–10% of all kinds of malignant tumors in the whole body, second only to uterine cancer in women, and has become the main cancer threatening women’s health. Fortunately, it has been proved that combination therapy of immunotherapy with radiotherapy, targeted technology, and even nanotechnology is active and effective in treating breast cancer, which provides a broader way of studying the treatment of breast cancer. Therefore, the purpose of our review is to study the value of immunotherapy in breast cancer treatment. Moreover, because the treatment of triple-negative breast cancer (TNBC) is difficult, we will discuss this in more detail in this article.

**Abstract:**

Breast cancer (BC) is a representative malignant tumor that affects women across the world, and it is the main cause of cancer-related deaths in women. Although a large number of treatment methods have been developed for BC in recent years, the results are sometimes unsatisfying. In recent years, treatments of BC have been expanded with immunotherapy. In our article, we list some tumor markers related to immunotherapy for BC. Moreover, we introduce the existing relatively mature immunotherapy and the markers’ pathogenesis are involved. The combination of immunotherapy and other therapies for BC are introduced in detail, including the combination of immunotherapy and chemotherapy, the combined use of immunosuppressants and chemotherapy drugs, immunotherapy and molecular targeted therapy. We summarize the clinical effects of these methods. In addition, this paper also makes a preliminary exploration of the combination of immunotherapy, radiotherapy, and nanotechnology for BC.

## 1. Introduction

Breast cancer (BC) comprises nearly 30% of cancers affecting women, and the mortality-to-incidence ratio reaches 15%. It is very heterogeneous, and, based on hormone receptor (ER and PR) and HER2 (ERBB2) status, can be clinically divided into luminal ER-positive and PR-positive, and further subdivided into luminal A and B, HER2-positive and triple negative breast cancer (TNBC) [1]. Compared with other malignancies, BC has a lower T cell infiltration level and smaller mutational burdens from an immunologic perspective. It is precisely because of the strong heterogeneity of the disease that related treatment is extremely difficult. Despite the decrease in BC mortality by 40% since 1989, patients suffering metastatic diseases still exhibit poor prognosis. In recent years, researchers have critically re-evaluated the effect of the immune system on BC progression and the therapeutic response and resistance, which provides new insight into immunotherapeutic treatment [2]. 

Tumors positive for HER2 respond well to HER2-targeted therapies, and this effect is partly mediated by immune effector mechanisms. Therefore, triple-negative breast cancer, which is considered to be the most immunogenic subtype, is emerging as a major area of development for immuno-oncology therapeutics. 

In terms of medication, atezolizumab, durvalumab, avelumab, etc. are common immunotherapeutic drugs for breast cancer, and a large number of experiments have proved their significant effects in the treatment of breast cancer. Furthermore, many clinical trials have tested many immunotherapies and other therapy combinations in all subtypes of breast cancer. Many of them have received good therapeutic effects. They effectively improved the objective response rate (ORR) of breast cancer patients and extended their overall survival (OS). Thus, in our article, we not only introduce the treatment of breast cancer with single immunotherapy but also discuss the combination of immunotherapy and other therapies for BC in detail, including the combination of immunotherapy and chemotherapy, the combined use of immunosuppressants and chemotherapy drugs, and immunotherapy and molecular targeted therapy. 

At the same time, although many studies are currently examining immune therapies for BC, still only a minority of patients appear to respond, and little is known about the underlying mechanisms of treatment efficacy. Thus, there is an unmet need to identify potential immuno-oncological prognostic and predictive markers to guide effective immunotherapies [3]. As a result, numerous studies have recently assessed the clinical value of immune modulation for BC patients, which are listed in our article.

## 2. Biomarkers of Immunotherapy Response in BC

### 2.1. Genomic Markers

Despite BC still being one of the most common cancers worldwide, the way people view it has changed considerably for its characterized molecular hallmarks. Among them, genomic markers closely correlate with mutation and the metastasis of the cancer. When it comes to immunotherapy of breast cancer, we will inevitably introduce genomic markers. The influential markers BRCA1, BRCA2, and PIK3CA are discussed below.

#### 2.1.1. BRCA1 and BRCA2

The BRCA1 and BRCA2 genes crucially regulate cell replication and growth. Under normal circumstances, BRCA1 and BRCA2 are typical tumor suppressor genes, which means that people with BRCA1 and BRCA2 genetic defects are highly susceptible to breast cancer [4]. Additionally, BRCA1 and BRCA2 are members of the important proteins that HRR, enabling fault-free repair of double-stranded DNA breaks, relies on. HRR mainly occurs in the S and G2 phases of the cell cycle. It is a DNA repair method to maintain the integrity of the genome and ensure the high-fidelity inheritance of genetic information. Without them, other pathways are utilized such as non-homologous end joining (NHEJ), leading to the accumulation of additional DNA amplifications or deletions, resulting in chromosomal instability [5]. Of course, the probability of people suffering from cancer will also vary due to the different mutation locations in BRCA1 or BRCA2 genes [6]. Therefore, exploring the causes of cancer caused by BRCA1/2 mutation from the perspective of mechanism is significantly helpful to evaluate the clinical risk of BRCA1/2 mutation carriers, and also helps to provide different immunotherapy programs for different mutation patients.

#### 2.1.2. PIK3CA

PIK3CA gene mutation is an oncogenic mutation of pan-cancer species, which leads to sustained activation of PI3K, uncontrolled proliferation of cells, and ultimately tumor formation. HER2-positive BC tumors show a good therapeutic effect for immunotherapy, while the early complete pathological response of HER2-positive BC can be better predicted by PIK3CA mutation [7]. However, it has been confirmed that tumors that carry a PIK3CA mutation exhibit an obviously lower pCR rate. Moreover, PIK3CA status is remarkably associated with HR status in trastuzumab- and lapatinib-treated groups [8]. Notably, although immuno-oncology and targeted inhibition of the PI3KCA pathway can be seen as non-redundant approaches, there is emerging evidence indicating that they can be combined to exploit potential synergy in triple-negative breast cancer [9]. Of course, some studies have pointed out that a large proportion of patients with solid tumors are eligible for immunotargeted combination therapy [10].

### 2.2. Immunohistochemical Markers

Immunohistochemical markers are proteins that indicate the difference between varying types of cancer. Specific cellular events (proliferation and cell death) are characterized by certain molecular markers. For BC, the extensively characterized markers are Ki67, ER, PR, HER2, and so on. In the histopathological diagnosis of some tumors, immunohistochemical markers play a key role. Different markers represent different tumor cells and have different treatment methods. Exploring markers can provide guidance for the treatment and prognosis of breast cancer.

#### 2.2.1. Ki67

The proliferative activity exhibited by tumor cells importantly reports the prognosis of cancer diagnosis. Immunostaining experiments show that Ki67 can only be observed in the cell nucleus for binding to the perichromosomal layer in dividing cells with active growth, which indicates that it can serve to evaluate cell proliferation as a useful marker [11]. However, recent studies indicate that Ki67 might have a valuable role in predicting the benefit of specific treatments in subtypes of breast cancer as well [1]. As a result, we would recommend applying Ki67 to immunotherapy of breast cancer.

#### 2.2.2. ER and PR

Research has shown that estrogen and progesterone are hormones that can promote the growth and proliferation of tumors. Through examination, it was found that the tumors of most BC patients always showed biological expression of estrogen and progesterone receptors [12]. Researchers have clearly determined the molecular mechanism regarding estrogen-to-receptor binding. Diffusing across the epithelial membrane of the breast, estradiol binds to the receptor in the nucleus. Hence, conformational change can be observed. The estradiol-to-receptor binding dimerizes with other receptors, and, as a result, estrogen-responsive genes are activated. Progesterone binds to nuclear receptors in a similar fashion to estrogen. ER has two forms, ERα and ERβ, with the former of clinical relevance. PR expresses in PRA and PRB. The currently adopted assays for PR in a clinical context bind to PRA and PRB or PRB only. Hormone receptors are unstable during the progression of a tumor. As ER and PR status may change for patients, a biopsy of the metastatic site for obtaining hormone receptor status before treatment is of great importance [13]. So far, although the response to immunotherapy is not common in ER-positive BC, a study has defined a new immune feature in PD-L1-negative ER-positive BC patients, which indicates that such BC patients are more likely to benefit from immune checkpoint and histone deacetylase inhibition [14]. These findings will provide new ideas for immunotherapy for BC patients.

## 3. Current Immunotherapy for BC

Originally considered to be immune silent, BC was hardly associated with immunotherapy in the past studies [15]. However, immunotherapy methods for this cancer have been developed rapidly in recent years since they were found useful for the treatment of BC. In immunotherapy, immunosuppressants targeting immune checkpoints are used to alleviate the response of patients and promote the antitumor effect of T cells, thus overcoming the immunosuppression of patients. To make antitumor immunotherapy effective, immune evasion is one of the main challenges to be overcome, because tumor cells can deploy abundant molecules and pathways for escaping immune surveillance as well as resisting the cytotoxic effect of host T cells. Therefore, we will introduce several common immunotherapy methods for breast cancer.

### 3.1. PD-1/PD-L1 Inhibitors

#### 3.1.1. Role and Significance of PD-1/PD-L1 Inhibitors

**Figure 1:**
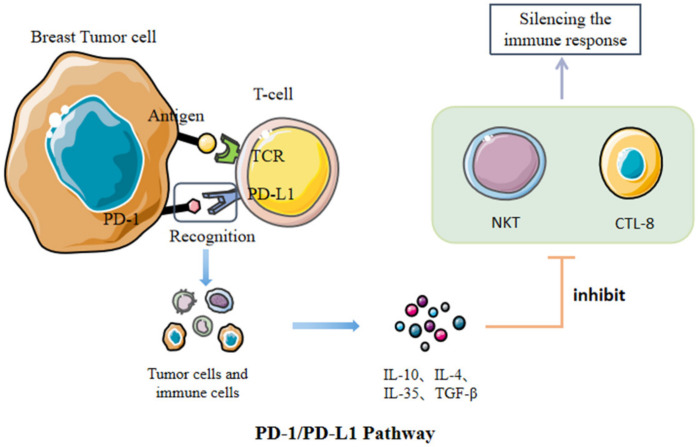
After the recognition of PD-1 and PD-L1, the immune escape of tumor cells can be realized by increasing the expression of inhibitory cytokines.

PD-1, primarily expressed on activated T cells’ surface, can combine with its ligand PD-L1, which presents expression on tumor cells and immune cells, to deprive T cells of killing ability. TCR receptors bind to specific MHC molecules of specific peptide chains, which is the first signal to activate T cells. However, PD-L1-to-PD-1 binding can suppress the function of lymphocytes and reduce the release of cytokines. It can also promote the apoptosis of lymphocytes (Figure 1). Immunosuppressive cells, occurring in BC tissues, take charge of releasing different negative regulatory factors and forming an immunosuppressive tumor microenvironment (TME) for preventing BC cells from being killed by CD8 + T cells. PD-L1-to-PD-1 binding is capable of limiting the activation and proliferation of tumor antigen-specific CD8+T cells [15]. Therefore, the function of CD8+T cells is inhibited, which may lead to the immune escape of tumors. Widespread acceptance as an immunosuppressive cytokine, interleukin-10 (IL-10), can diminish the antitumor immune response, thereby promoting tumor escape in the TME [16]. When bound to PD-1, PD-L1 can promote IL-10 expression, enhancing immunosuppress and thus leading to the escape of the tumor [17]. PD-1/PD-L1 inhibitors are capable of blocking PD-L1-to-PD-1 binding and reducing the immune escape phenomenon of tumors. Of course, we can also directly target the PD-L1 gene that modifies the tumor in order to significantly reduce or even inhibit its expression, thus avoiding the immune escape of the tumor [18]. At present, most of the immune drugs used to treat breast cancer target PD-1 or PD-L1.

#### 3.1.2. Expression of PD-L1

Many cancers present the expression of PD-L1, including renal cell carcinoma, ovarian cancer, etc. [19]. PD-L1 expression is a biomarker that predicts the efficacy exhibited by PD-1/PD-L1 inhibitors specific to other tumors [20]. In TNBC, trials have shown the greater possibility of PD-L1 + patients being positively treated by immunotherapy [21]. Furthermore, according to one study, in advanced TNBC, the response rate is 5–23% and higher PD-1 and PD-L1 mRNA expressions report better prognosis [22]. However, standard PD-L1 detection analysis is not available currently, and common immunohistochemical analysis is conducted on two platforms (Dako Link 48 and Ventana BenchMark), which involves 22c3, sp263, and sp142. Ventana sp142 analysis assists in examining the predictive effect of PD-L1 expression in the palacion130 study, investigating the expression of PD-L1. Furthermore, it can identify PD-L2 staining of any intensity, with its sub studies focusing on evaluating the predictive power exhibited by different PD-L1 assays as well [17]. Therefore, the sp142 test has been approved as an auxiliary diagnostic device specific to TNBC patients who chose atezolizumab treatment by the US Food and Drug Administration (FDA) since 8 March 2019 [17]. In addition, some researchers also found it a good alternative to detect the PD-L1 mRNA expression for TNBC. For example, to evaluate for the presence of PD-L1 in breast cancer, a study based on TCGA RNA sequencing data and laser capture microdissection was performed. Analysis showed that, compared with non-TNBC (*n* = 716) samples, the difference in PD-L1 expression in TNBC (*n* = 120) was significantly higher (*p* < 0.001). Furthermore, after RNA extraction, qRT-PCR confirmed the existence of PD-L1 mRNA in all their samples, and two of the three tumors (LCM1 and LCM4) with the highest PD-L1 mRNA level are TNBC [23]. Therefore, a combination of methods is ideal for detecting PD-L1 expression.

#### 3.1.3. PD-1/PD-L1 Inhibitor Administration and Effect Detection

The molecular structure presents small differences for different anti-PD-1 and anti-PD-L1 antibodies that are applied clinically. Specifically, pembrolizumab and nivolumab are a humanized IgG4 monoclonal antibody and a fully human monoclonal IgG4, respectively. Durvalumab and avelumab are a fully human monoclonal IgG1-κ and IgG1-λ, respectively. Studies have not yet confirmed whether these differences are capable of translating into different clinical efficacy or toxicity profiles. In general, according to the current data, all antibodies that target the PD-1 axis exhibit roughly similar clinical efficacy and toxicity [21].

Anti-PD-1/PD-L1 immunotherapy has great potential as a strategy for treating different cancers, such as BC (Table 1). However, the effectiveness can be weakened under the impact of its primary or acquired resistance when the therapy progresses [22]. Currently, many clinical trials using pembrolizumab and atezolizumab are underway [23]. Anti-PD-1 or anti-PD-L1 drugs are employed as monotherapy for treating different BC subtypes, with preliminary activity being shown in five phase 1 and phase 2 trials. The proportion of patients who obtained objective responses was between 5% and 24%, similar to other cancers, with many responses being persistent. The keynote-012 test focused on evaluating the safety and efficacy exhibited by pembrolizumab in metastatic TNBC. Results showed that it was related to PD-L1 expression, with 19% of the 27 patients who were evaluated achieved overall efficacy through immunohistochemical detection and anti-PD-L1 22c3 antibody detection. In contrast, the keynote-086 test, which examined the safety of pembrolizumab in metastatic TNBC treated previously, was not related to PD-L1 expression and reported that the overall objective response rate was 21%. Other immunotherapy trials found some durable response besides the short-lived reactions common to chemotherapy. The median reaction duration was 10.4 months (ranging from 4.2 to 19.2), and some reactions were still in progress at the data cutoff [21]. However, due to the mild response specific to PD-1/PD-L1 monotherapy, research on TNBC should adopt combination therapy as proposed above [15]. Nivolumab is usually used in combination with ipilimumab (a kind of CTLA-4 inhibitor) to treat breast cancer. Recently, an experiment was conducted to evaluate the effect of nivolumab and ipilimumab in the combined treatment of unresectable or metastatic metallic BC. In this experiment, the objective response rate of this treatment can reach 18%, though adrenal insufficiency was observed in all responders. Responses occurred in tumors with low tumor mutational burden, low PD-L1, and absent TILs [24].

### 3.2. CTLA-4 Inhibitors

**Figure 2:**
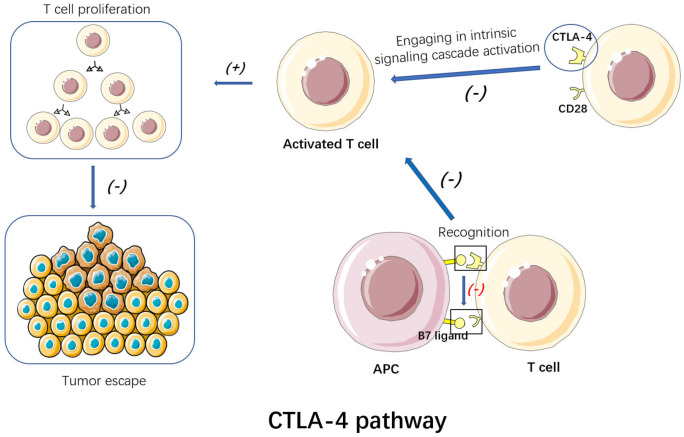
By blocking CTLA-4, CTLA-4 inhibitors can destroy the intrinsic signaling cascades. Thus, T cell proliferation is promoted, avoiding the immune escape of the breast tumor.

CTLA-4 (cluster of differentiation 152, CD152) is a receptor on activated T cells’ surface, and belongs to the family of immunoglobulin-related receptors taking charge of T cell immune regulation [28] (Figure 2). According to further research, CTLA-4 engagement resulted in intrinsic signaling cascade activation in T cells. It competes with CD28 receptors to bind to B7 ligands (B7-1/CD80 and B7-2/CD86) on antigen-presenting cells (APCs). However, CTLA-4 receptors are capable of binding to B7 ligands at a weaker surface density with higher affinity, hence performing better than CD28 receptors in the binding [20]. CTLA-4 is upregulated soon after the activation of T cell, providing negative feedback to CD28 co-stimulation via binding with CD80/CD86 and restricting the activation of T cells in the immune response priming phase [29]. The decline of T cell proliferation promotes the escape of the breast tumor. Blocking of CTLA-4 will destroy the cascade, yielding numerous lymphocytes [17]. CTLA-4 can interfere with TCR signal by interacting with PP2A and SHP2. At the same time, CTLA-4 binds with PI3K, leading to AKT phosphorylation and causing the inactivation of proapoptotic factor BAD. It can also regulate antiapoptotic factors, Bcl-xL and Bcl-2, which play a key role in immune tolerance. CTLA-4 is also an inhibitory receptor on the surface of FOXP3+ Treg cells, which are important components of the tumor microenvironment (TME). They accumulate at tumor sites and in the peripheral circulation of patients with cancer. It is generally believed that Treg cells interfere with antitumor immunity and thus promote tumor progression. Thus, FOXP3+ Treg may serve as a therapeutic target for promoting antitumor immunity [30]. CTLA-4 inhibitors avoid immune escape of breast tumor and achieve the goal of treatment by blocking the CTLA-4 pathway.

In general, a CTLA-4 inhibitory signal pathway can be observed between the antigen-presenting cells and lymphocytes to prevent excessive activation of lymphocytes, leading to immunity-related damage. The blocking of CTLA-4 will destroy the cascade reaction and generate many lymphocytes. While therapy adopting the CTLA-4 antibody is capable of inducing CD8+T cell expression as found. Anti CTLA-4 is limited to the intracellular signal pathways, like the regulation pathway of a cell cycle, and mechanism overlapping will not occur. In addition, CTLA-4 shows an association with the initial T cell activity stage, but PD-1/PD-L1 presents expression on T cells activated at a later stage. Recently, many CTLA-4 inhibitors (such as ipilimumab and tremelimumab) or their combinations have been shown to exert their functions in solid tumors. The FDA has approved the Ipril monoclonal antibody to improve the survival rate of advanced metastatic melanoma, while moderate antitumor activity is generated in TNBC [17].

CTLA-4 are also constitutively expressed on regulatory T cells (Treg cells), which helps to regulate immunological self-tolerance. An anti-CTLA-4 antibody may be capable of killing tumor-infiltrating effector Treg cells or lowering the suppressive activity. However, anti-CTLA-4 monotherapy presented limited or no efficacy; hence, other therapeutic strategies shall be taken into account. Combinations within a step and across steps of the cancer-immunity cycle may be clinically beneficial. Immune checkpoint blockade (ICB) considering other immune checkpoints has made progress in TNBC as well. Furthermore, based on preclinical and clinical data, cancer vaccines carrying anti-CTLA-4 antibody have been applied [31]. 

In some clinical trials, a tumor vaccine is combined with an anti-CTLA-4 monoclonal antibody for treating TNBC. It is reported that, compared with the single vaccine or anti-CTLA-4 monoclonal antibody, immunotherapy combining the MUC1 mRNA nanovaccine, another kind of tumor vaccine, and anti-CTLA-4 monoclonal antibody is capable of effectively suppressing TNBC growth and significantly increasing the tumor-infiltrating CD8+T cell number. In TNBC, a higher level of tumor-infiltrating lymphocytes extend OS and improve the objective response rate. Nevertheless, immunosuppressive molecule expression and inhibitory immune cell infiltration into the TME significantly suppress the tumor-killing effect medicated by cytotoxic T lymphocyte (CTL). The combination treatment is assumed to be capable of reducing immunosuppressive TEM, normalizing tumor vasculature, and inhibiting tumor-promoting signaling pathways for enhancing antitumor CTL activity and more deeply inhibiting TNBC growth. The combination treatment can also enhance CD8+ T cell infiltration into tumor sites, and improve the antitumor cytotoxic T-lymphocyte activity according to the study [31].

### 3.3. Globo H Antitumor Glucose Vaccine

**Figure 3:**
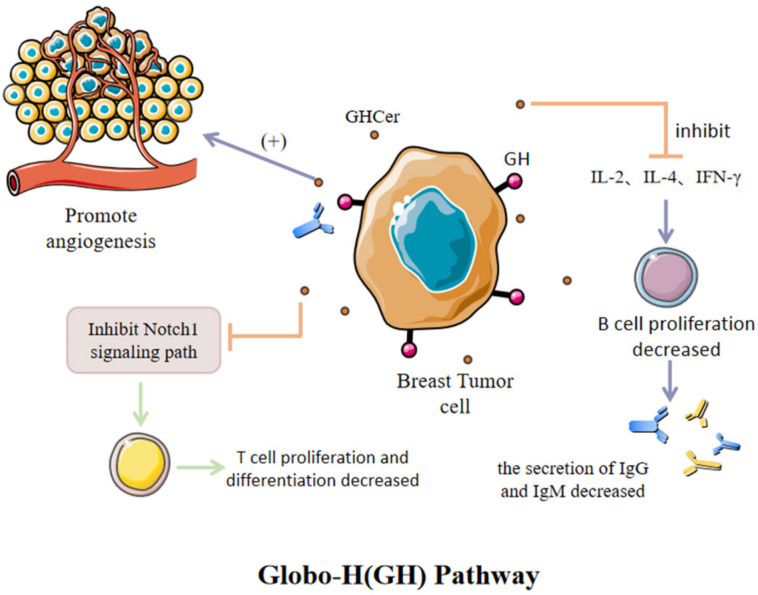
GHCer, which exists in the microenvironment of BC, can promote tumor development by promoting vascular proliferation, inhibiting Notch1 signaling pathway and inhibiting the release of specific antibodies.

Globo H (GH), a kind of hexasaccharide (Fucα1–2Galβ1–3GalNAcβ1–3Galα1–4Galβ1–4Glc) obtained from the human BC cell line MCF-7 as a ceramide-linked glycolipid, is a representative TACA (tumor-associated carbohydrate antigen) (Figure 3). It exhibits high expression on BCs. GH can greatly help to regulate the tumor microenvironment and promotes tumor progression via multiple mechanisms [32]. Globo H Ceramide (GHCer) exists in the TME, and can be absorbed via tumor-infiltrating lymphocytes. Several studies have shown that GHCer may mediate tumor immune escape by inhibiting Notch1 signal transduction [33]. GHCer can also inhibit IL-2, IL-4 and IFN-γ to reduce the proliferation of CD19+B lymphocytes and human peripheral blood mononuclear cells (PBMC). As a result, the secretion of IgM and IgG, especially IgM, are greatly reduced. GHCer can also be incorporated into endothelial cells to promote angiogenesis [34]. According to this pathway, we expect to use the GH antigen vaccine to induce IgG antibody and T cell-dependent response, with the aim of blocking the immunosuppression of the tumor. However, several challenges had to be overcome before th e production of vaccines which are suitable for clinical use. In general, sugar chains often induce weak immunogenic responses, and the antibodies they engender typically have low affinity [35]. To overcome these challenges, researchers have developed multicomponent vaccines, including the glycan antigen, a carrier protein (e.g., keyhole limpet hemocyanin (KLH)), and an immunological adjuvant (e.g., QS-21) [35,36,37]. For example, an investigational immune stimulant (vaccine) comprising the Globo H hexasaccharide epitope is linked to the carrier protein keyhole limpet hemocyanin (KLH). The mechanism of action of KLH facilitates a more vigorous immune response given the weak antigen. Globo H induces anti-Globo H IgG and IgM that recognize Globo H expressed on the surface of 60–80% of breast cancer cells. The IgM antibodies recruit complements to attack the tumor cells, while the IgG antibodies guide NK cells to destroy the tumor [38].

In BC, Globo H is overexpressed in about 60% of ductal, lobular, and tubular adenocarcinomas [39]. Globo H was specifically expressed in breast cancer stem cells (BCSCs), but the expression frequency (20% (BCSCs) vs. 61% (non-BCSCs)) and expression level (9.7–71.0% (BCSCs) vs. 14.3–75.2% (non-BCSCs)) were lower compared with those in non-BCSCs [40]. This variability makes the Globo H antigen an ideal target for antitumor immunotherapy.

Human umbilical vein endothelial cells (HUVECs) can absorb Globo H ceramide (GHCer) from vesicles exfoliated from breast cancer cells [41]. GHCer, as an angiogenic factor, can significantly promote the migration and vascularization of HUVECs. A study found rapid proliferation of breast cancer cells with high Globo H expression in vivo as compared to cells with low or negative Globo H expression, and the tumor tissue contained higher blood vessel density in comparison with that in normal tissue, proving that Globo H can promote tumor angiogenesis and enhance tumorigenicity [34]. At present, the antitumor vaccines OBI-822 and OBI-833 designed and synthesized with Globo H as the target antigen have entered phase I, II, and III clinical trials for BC. Among them, the Globo H-KLH/QS-21 (OBI-822, alias Adagloxadsimolenin) antitumor vaccine has been confirmed to have good immunogenicity and tolerance in phase I clinical trials of advanced breast cancer. Twenty-seven patients with metastatic breast cancer received the Globo HKLH/QS-21 vaccine. Five consecutive vaccinations were performed at the 1st, 2nd, 3rd, 7th, and 19th weeks, and the IgG and IgM antibody titers of anti-Globo H in peripheral blood were detected regularly. It was found that the vaccine was well tolerated by patients, and local skin reactions and mild flu-like symptoms were the main side effects. Sixteen patients developed markedly elevated titers of IgM antibodies, showing specific recognition of the Globo H antigen [42]. This result encourages further exploration for the development of Globo H antitumor vaccines. 

### 3.4. Breast Cancer Stem Cells

Tumors are composed of heterogeneous cell groups, in which an undifferentiated cell that can produce differentiated cells and constitute most of the few subgroups of tumors are found, called cancer stem cells (CSCs). As revealed by many studies, tumor stem cells (CBCs) are capable of being isolated from different tumors [43], including breast cancer. These tumor stem cells found in breast cancer are called breast cancer stem cells (BCSCs) [44].

Meanwhile, breast cancer stem cells can promote angiogenesis by differentiating endothelial cells and secreting angiogenesis factors. In addition, the multidrug resistance genes and drug-excreted transporter expressed in breast cancer stem cells equip various conventional chemotherapy drugs with drug resistance. Therefore, designing treatment interventions for cancer stem cells will help improve the survival rate of patients [45].

In the treatment of breast cancer, conventional therapeutic agents kill most breast cancer cells without eliminating BCSCs, and sometimes even increase the proportion of BCSCs in breast tumors. Therefore, it is necessary to develop specific and effective methods to eliminate BCSCs.

On the one hand, with anti-BCSC drugs’ clinical transformation hindered by many problems, such as instability, low bioavailability, and off-targeting effects, a team study has proved that the nanopharmaceutical delivery system (NDDS) has the potential to overcome the shortcomings of anti-BCSC drugs by providing site-specific delivery and enhancing the stability and bioavailability of delivery agents [46].

On the other hand, targeting BCSCs can improve the therapeutic effect [47]. Additionally, targeting the lncROPM-PLA2G16 signaling axis may also be a new treatment strategy for breast cancer patients [48].

Furthermore, it cannot be ignored that the great success of immunotherapy has brought new hope to countless cancer patients. The study found that a small portion of CSCs did not function in most current therapies and affected metastasis and recurrence, which clearly shows that the ideal target of immunotherapy is antigens expressed in CSC, which played a key role in its function. These antigens are called CSC cancer antigens. In fact, their immune targeting will enable the eradication of CSC, thus eliminating tumor sources [49].

In short, BSCS remains a good candidate for cancer immunotherapy and still has great future research potential.

## 4. Immunotherapy Combined with Other Therapies

### 4.1. Immunotherapy Combined with Chemotherapy

**Figure 4:**
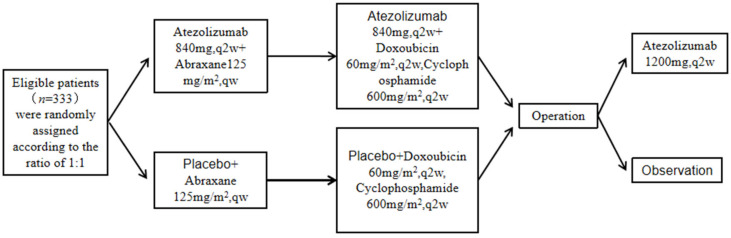
Experimental flow of IMpassion031.

TNBC is characterized by strong invasive ability, poor prognosis, and few therapeutic targets. With the renewal of neoadjuvant therapy concepts and the continuous expansion of indications, patients with TNBC have also become a relatively preferred target population for neoadjuvant chemotherapy [1]. IMpassion031 compared efficacy and safety of atezolizumab versus placebo combined with nab-paclitaxel followed by doxorubicin plus cyclophosphamide as neoadjuvant treatment for early-stage TNBC (Figure 4). In their study, patients of newly-diagnosed stage II-III TNBC with tumor diameter >2 cm, aged ≥18 years, were randomly divided into two groups, with the experimental group containing 165 cases and the control group containing 168 cases. The experimental process is as follows:

The primary evaluation indexes were pCR in the intention-to-treat (ITT) population and PD-L1-positive patients, and the secondary evaluation index was EFS and safety. The results showed that the atezolizumab plus chemotherapy group had a similar median follow-up time to the placebo plus chemotherapy group, but the former group had a higher number of patients achieving a pathological complete remission compared with the latter group. Severe adverse events (AEs) occurred in 30% and 18% of the atezolizumab group and placebo group, respectively, and included neutropenia, fever, and rash [50]. The IMpassion130 study demonstrated for the first time that the PD-L1 inhibitor atezolizumab combined with nab-paclitaxel in treating advanced TNBC can significantly enhance the PFS and overall survival [51], confirming the advantages of immunotherapy in treating TNBC patients [50].

### 4.2. PD-1/PD-L1 Inhibitors Combined with Trastuzumab

Human epidermal growth factor receptor 2 (HER2) belongs to the HER family (EGFR, ErbB), and is a receptor tyrosine kinase. Overexpressed in approximately 20% of all BCs, it is the main drug target for targeted therapy of BC [52,53]. Though trastuzumab, a humanized anti-HER2 monoclonal antibody approved for treating HER2-positive BC in 1998, acts as the first targeted therapeutic drug specific to BC targeting HER2 [54], its general resistance in the clinic has become a great challenge for targeted therapy. Some studies have shown that ICIs can enhance the therapeutic effect of molecular target inhibitors [21]. Therefore, the combination of ICIs and molecular target inhibitors has become a new way to treat BC. 

Pembrolizumab is a humanized monoclonal IgG4-j antibody with strong selectivity, capable of blocking the interaction of PD-1 with its ligands, PD-L1 and PD-L2 [55]. A trial (NCT02129556) studied the effect of trastuzumab combined with pembrolizumab in detail. The phase 1b study held the primary endpoint of confirming the dose-limiting toxicity incidence and the suggested dose in phase 2. The phase 2 study held the primary endpoint of determining the percentage of PD-L1-positive patients who achieve an objective response. The experiment proved that pembrolizumab combined with trastuzumab was safe in patients suffering PD-L1-positive, trastuzumab resistance, and advanced HER2-positive BC, showing activity and lasting clinical benefits. This experiment also determined the correct dosage of medication [21,56]. 

Durvalumab is a monoclonal antibody for PD-L1, with activity in various tumor types such as urothelial cancer, non-small cell lung cancer (NSCLC), melanoma, head and neck cancer, and gastroesophageal cancer, which can be combined with trastuzumab in treating HER2-positive BC [56]. The CCTGIND.229 (NCT02649686) phase 1 trial examined the treatment combining durvalumab with trastuzumab in 15 metastatic HER2-positive BC patients. All the participants had undergone wide HER2-targeted treatment previously. As revealed by related studies on advanced-stage BC, it is suggested to focus on investigating the application of checkpoint inhibitors in early treatment and conducting enrichment therapy for patients having a larger number of immunogenic tumors (e.g., PD-L1 expression and stromal T cell infiltration occurred before treatment). Patients having heavily pretreated HER2-positive PD-L1-negative MBC did not present obvious clinical activity. Furthermore, during the treatment period, all the experimental participants also experienced adverse reactions, such as headache, fatigue, nausea, cough, anorexia, back pain, and constipation, which are related to this treatment scheme too [21,57].

Trastuzumab can also be combined with atezolizumab for treating patients suffering HER2-positive metastatic BC. The trial KATE2 (ClinicalTrials.gov, accessed on 26 September 2016; Identifier: NCT02924883) assessed the safety and efficacy exhibited by the combined treatment of atezolizumab and trastuzumab emtansine. Relative to the trastuzumab emtansine + placebo group, patients in the trastuzumab emtansine plus atezolizumab group appeared in their ATA response to have a higher chance of suffering from eye disorders, endocrine disorders, gastrointestinal disorders, pyrexia, and respiratory infection, and of possessing prolonged PFS [58,59] (Table 2).

### 4.3. PD-1/PD-L1 Inhibitors Combined with Molecularly Targeted Therapies

#### 4.3.1. Immunotherapy Combined with Poly(ADP-ribose) Polymerase (PARP) Inhibitors

Recently, PARP inhibitors were suggested to function in antitumor immunity. There is potential synergy between PARP inhibitors and immune checkpoint inhibitors [60].

The concept of synthetic lethality promotes the development of PARP inhibitors in cancers possessing the defect of homologous recombination repair (HRR), especially those with biallelic loss of BRCA1 and BRCA2 [61]. According to the literature, additional PARP inhibition could promote cross-presentation and modify the immune microenvironment for alleviating resistance as well as strengthening the efficacy exhibited by ICB therapy [62].

PARP-based therapies work through the inhibition of single-strand DNA repair, leading to DNA damage, and increased tumor mutational burden, making the tumor a more attractive target for immunotherapy. Of the solid tumors reviewed, efficacy in the combination of PARP inhibition and immunotherapy has been demonstrated in breast cancer, predominately in BRCA-mutated tumors [63].

Olaparib and talazoparib, as PARP inhibitors, serve as monotherapies for treating metastatic BC patients [21]. During the follow-up period of the MEDIOLA trial (NCT02734004), the combination therapy was well tolerated, with no evidence showing a drug–drug interaction between olaparib and duvalizumab [59]. The trial KEYNOTE-162/TOPACIO (NCT02657889) also confirmed that adverse immune event incidence did not increase in combination treatment of niraparib and pembrolizumab compared with treatment of pembrolizumab alone. The condition of many patients has been alleviated to some extent [64]. Many experiments show that treatment combining a PARP inhibitor with an anti-PD-1 antibody exhibits a tolerable safety profile for patients suffering from advanced or metastatic TNBC and a stronger antitumor activity, regardless of the BRCA mutation status. This combination therapy also contributes to a different therapeutic approach to treating BC (Table 3).

#### 4.3.2. Immunotherapy Combined with Inhibitors of Cyclin-Dependent Kinase 4 (CDK4) and CDK6

For CDK4/6 inhibitors, representing a new generation of therapeutics, it will be critical to understand the cellular mechanisms by which they act. Today, far more specific inhibitors that target CDK4 and CDK6 exist. These have more limited toxicity, which allows for their broad use in treating a variety of neoplasms [70]. In fact, the primary action mechanism of CDK4/6 inhibitors is to inhibit the phosphorylation of retinoblastoma (RB) proteins for inducing cell-cycle arrest in G1-phase [71]. This ability to induce DNA damage now provides a rationale to better predict responsive tumor types and effective combination therapies [72].

According to recent research, small-molecule inhibitors of CDK4/6 have been used with great success in the treatment of hormone receptor-positive breast cancers, and are in clinical trials for many other tumor types [73].

Notably, three CDK4/6 inhibitors, namely, palbociclib, ribociclib, and abemaciclib, are currently approved for clinical use in the United States for use in combination with endocrine therapy for women with advanced hormone receptor positive breast cancer [74]. Of these three inhibitors, the FDA approval of palbociclib used with the aromatase inhibitor letrozole for breast cancer treatment highlights long-sought success [75]. Taking all the mechanisms and factors into consideration, the focus of future studies should be on the development of biomarkers so that patients who are likely to be resistant to CDK4/6 inhibition can initially be given alternative methods of treatment [76].

Currently, CDK4/6 inhibitors are being explored in combination with other agents, including targeted therapies, immunotherapy, and chemotherapy, bringing new hope to breast cancer patients.

#### 4.3.3. Immunotherapy Combined with Angiogenesis Inhibitors

Breast cancer is highly resistant to the different types of chemotherapy, so many teams are researching and developing new alternative strategies. One of these strategies that has also attracted much attention in recent years is to suppress tumor growth by inhibiting angiogenesis. In breast cancer, the main angiogenesis factors are the VEGF, Ang-1/Te-2, PDGF, and bFGF pathways [77]. Based on these pathway targets, anti-angiogenic drugs can be developed, of which the most frequently used is the Bevazhu monoclonal antibody, a recombinant humanized monoclonal antibody that targets all known VEGF-A subtypes [69].

Small molecules with anti-angiogenic activities are the most commonly used antiangiogenic agents, such as Bisindole-PBD (5b) and rhubarb. Data from one team proved that 5b inhibited the expression of VEGF at transcription and post-recorded levels, further regulating BC cell proliferation [66]. Furthermore, one study identified that emodin can greatly increase the expression of SerRS in TNBC cells, thus inhibiting VEGFA transcription and effectively inhibiting vascular development and blocking tumor angiogenesis in mice carrying TNBC [67]. Additionally, as a small molecular kinase inhibitor, Cabozantinib (XL184) is effective in MET and VEGF2 and many other receptor tyrosine kinases. Experiments confirm XL184 as a promising drug that inhibits the angiogenesis and metastasis of cancer tumor with MET and VEGFR signaling disorders, and does not increase the burden caused by lung tumors [65]. Finally, in-depth research shows that SAL interrupts HIF-1α/VEGF signaling for inhibiting angiogenesis and BC growth induced by VEGF, and is a promising antiangiogenic agent for treating BC [68].

To date, testing of combinations of anti-angiogenic treatment with immunotherapy has reached phase III development and includes trials in hepatocellular carcinoma, advanced renal cell carcinoma, NSCLC, and ovarian cancer patients. Consequently, clinical development of anti-angiogenic therapy was a complete success. However, the benefits are limited and we cannot select patients likely to respond to this therapy, which still remains a challenge [78].

### 4.4. Immunotherapy Combined with Radiotherapy 

New data have emerged that show the synergy of radiotherapy with immunotherapy in controlling or eradicating cancer [79]. Some researchers have even proposed a model in which irradiation and immunotherapy synergize to exert more potent local effects on the irradiated tumors [80]. 

The combination of radiotherapy and immunotherapy serves to combat drug resistance and strengthen efficacy. First, it enhances IFN-γ. The released T cells and T cell priming promote the destruction of tumor cells. In addition, experiments have proved that pembrolizumab treatment after radiotherapy can also improve the OS of NSCLC patients [22]. Second, there was an abscopal effect in the trials of the therapy combining cancer radiotherapy and immune checkpoint therapy [21]. Moreover, another study confirmed that D-mannose facilitates immunotherapy and radiotherapy of triple-negative breast cancer via the degradation of PD-L1 [81]. 

In summary, a promising breast cancer treatment is that of combining immunotherapy with radiotherapy.

### 4.5. Immunotherapy Combined with Nanotechnology 

Although breast cancer immunotherapy has many advantages, its clinical application has some limitations. For instance, when immunostimulants are insufficiently delivered to the immune cells and immune system modulation is out of control, there will be poor immune responses, giving rise to autoimmunity and nonspecific inflammation [82]. TME, weak immunogenicity, and off-target toxicity also restrict the wide application of immunotherapies [83]. Mounting evidence shows that nanotechnology may solve the problems faced in immunotherapy of breast cancer. The rapid development of nanotechnology is accompanied by many approaches of imaging, detection, diagnosis, and delivery of chemotherapeutic drugs to the tumor site [84]. Nanoparticles have many advantages, such as stronger biocompatibility, weak side effects, prolonged half-life, good biodistribution, and regulated stimuli-responsive release regarding therapeutic agents; hence, they are proper candidates for the immunotherapy. Advanced nanoparticles can also enhance the immunotherapy efficiency and greatly weaken their toxic side effects [82]. Therefore, nanotechnology combined with immunotherapy is expected to become a new treatment for breast cancer. TiDMSN exhibits a strong specificity for enriching phosphoproteins. Some experiments used TiDMSN for in situ phosphorylation protein enrichment and combined ICD and ICB (PD-L1 antibody) immunotherapy for BC. In addition to an improvement in therapeutic effect of ICD strategy, these experiments found an accumulation of TiDMSN in the primary tumor site but nanoparticles were not significantly migrated to distant tumor sites, indicating that the side effects resulting from nanoparticles were small in the treatment. TiDMSN can also significantly enhance the therapeutic effects and hemolytic effect [85]. Furthermore, PS3D1@DMXAA treatment (a novel strategy that combines chemotherapy and immunotherapy for modulating the TME via systematic and concurrent delivery of the chemotherapeutic agent SN38 and the STING agonist DMXAA into the tumor via triblock copolymer nanoparticles) induced systemic antitumor immunity. PS3D1@DMXAA can also trigger the highest secretion of IFN-γ and TNF-α in the serum, an essential mechanism that explained the successful antitumor effect [83]. In addition, some experiments show the surprising positive therapeutic effects of the combination of nanotechnology and immunotherapy. However, this treatment method also has its own defects. Some nanomedicine is based on inorganic material open vascular endothelial channels, while promoting the metastasis of cancer cells in blood vessels, which may increase the metastasis risk [84]. Lysosomal degradation regarding nanotherapeutics also restricts nanomedicine from being widely applied in anticancer therapy [86] (Table 4).

Unfortunately, these treatments have only been tested on animals. Whether immunotherapy combined with nanotechnology has potential toxic side effects is unknown to us and remains to be discovered. Because of many unknown factors, there is still a long way to go in terms of the clinical application of immunotherapy combined with nanotechnology for breast cancer treatment.

### 4.6. Combination of Immunodrugs

The diversity in positive/negative feedback loops of the immune system and the intertwined regulatory mechanisms provide abundant potential strategies for enhancing the modest single-drug activity exhibited by antibodies that target PD-1 and PD-L1. These strategies can be divided into three categories: strategies aimed at increasing cancer antigen presentation, strategies aimed at enhancing immune cell penetration into the tumor microenvironment, and strategies aimed at enhancing effector cell activity. These strategies are implemented practically by the development of vaccines, targeting of chemokines responsible for immune trafficking regulation, activation of co-stimulatory molecules, and inhibitory components capable of inhibiting the T cell receptor signaling axis to genetically engineered T cells and multifunctional molecules. Clinically, the combination of anti-CTLA-4 and anti-PD-1 drugs in treating metastatic melanoma is very effective, but it exhibits greater toxicity in relation to the single drug, confirming the successful combination of immunotherapeutic drugs. The anti-PD-1/MEK inhibitor selumetinib combination can also induce effective antitumor immune responses in MMTV neu (HER2 BC mouse model) and TNBC tumor models [87].

Generally speaking, immunodrugs are promising for the treatment of breast cancer, but there is still a long way to go.

## 5. Conclusions and Prospects

People’s views on breast cancer have changed greatly because of its unique molecular characteristics, especially TNBC. Among these features, genomic markers are closely related to mutations and cancer metastasis, with BRCA1, BRCA2, and PIK3CA being previous impact markers. Under normal conditions, BRCA12 is a typical tumor-suppressor gene. However, their mutations are associated with cancer, especially breast cancer, and the cancer risk varies with the mutation position in BRCA1 or BRCA2 genes. Pik3a is an oncogenic mutation of pan-cancer species, and the pathogenic mutation of PIK3CA leads to uncontrolled proliferation of cells and eventually tumor formation. In the histopathological diagnosis of some tumors, immune markers play a key role, including Er, PR, and Ki67, which are believed to have a valuable role in predicting benefits in subtypes of breast cancer as well.

The current research progress shows that immunotherapy for TNBC is mainly through three pathways, namely, the PD-1/PD-L1 pathway, the CTLA-4 pathway, and the Globo H pathway. Overcoming the patient’s immunosuppression is a prerequisite for successfully carrying out these three therapies. The most significant one is PD-1/PD-L1 inhibitor, but CTLA-4 inhibitor and Globo H inhibitor are also important. Recently, the research on several combinations of these inhibitors has also made some progress.

BCSCs are the target of immunotherapy for breast cancer. Clinical analysis of BCSCs in breast tumors found a correlation between the proportion of BCSCs and poor prognosis. In breast cancer, CSCs are thought to contribute to malignant progression. This indicates that targeting breast cancer stem cells (BCSCs) can improve the therapeutic effect. However, there are objective differences in the frequency and phenotype of BCSCs in varying breast cancer cell lines and patients, and the regulatory mechanism of BCSCs is still unclear.

To apply PD-1/PD-L1 inhibitors for treating BC patients, the further combination therapy of chemotherapy or targeted drugs is the new focus of TNBC research. The ipassion130 study proved for the first time that, in treating advanced TNBC, the PD-L1 inhibitor atezolizumab+nab paclitaxel is capable of significantly enhancing the PFS rate and OS rate of PD-L1-positive patients, confirming the superiority exhibited by immunotherapy in the neoadjuvant treatment of TNBC patients. Apart from those mentioned above, there are more combined therapies for BC. For instance, trastuzumab is a humanized anti-HER2 monoclonal antibody, and was used for treating HER2 positive BC in 1998. It can also be combined with pembrolizumab or durvalumab for treating HER2-positive metastatic BC patients. Pembrolizumab is also capable of blocking the interaction of PD-1 with its ligands PD-L1 and PD-L2. Durvalumab can also be combined with trastuzumab in treating HER2-positive BC. Combining PARP inhibitor with anti-PD-1 antibody exhibits tolerable safety together with good antitumor activity in advanced or metastatic TNBC patients. This combination therapy also contributes to a different therapeutic method for treating BC. Meanwhile, the combination of ICIs and molecular target inhibitors has become a new way to treat BC.

Extensive efforts to establish effective therapies are currently under way—suppressing tumor growth by inhibiting angiogenesis has attracted much attention in recent years. At the same time, radiotherapy has a potential synergy with immunotherapy, and so does chemotherapy. CDK4/6 inhibitors and PARP are being explored in combination with immunotherapy as well, bringing new hope to breast cancer patients. Notably, some researchers believe that immunotherapy combined with nanotechnology can also enhance the immunotherapy efficiency and greatly weaken their toxic side effects.

Despite these benefits, certain challenges cannot be overlooked. Firstly, the general resistance of HER2 in the clinic has become a great challenge for targeted therapy. Secondly, the benefits are limited and we cannot select patients likely to respond to a specific therapy. Thirdly, clinical application has some limitations. For instance, when immunostimulants are insufficiently delivered to the immune cells and immune system modulation is out of control, there will be poor immune responses, giving rise to autoimmunity as well as nonspecific inflammation. Finally, some of these treatments have only been tested on animals, which means that it is still unknown to us, and remains to be discovered, whether they have potential toxic side effects.

In conclusion, some advances in immunotherapy of BC have been made, but we still believe these combination treatments are bound to lead to breakthroughs in the future.

## Figures and Tables

**Table 1:** Treatment outcome of clinical trials for PD-1/PD-L1 inhibitors.

Drugs	Target	Condition	Objective Response Rate	Overall Survival (Months)	Progression-Free Survival (Months)	Adverse Events	Reference
Atezolizumab	PD-L1	mTNBC	10%	17.6	1.4	Pyrexia, fatigue, nausea	[25]
Durvalumab	PD-L1	mBC	-	21.7	2.7	Pneumonia, hepatitis	[26]
Avelumab	PD-L1	mBC	3%	8.1	5.9	Fatigue, nausea, constipation, hypothyroidism	[27]
		TNBC	5.2%	9.2	5.9		
Ipilimumab and Nivolumab	CTLA-4 and PD-1	MpBC	18%	12	2	Adrenocortical hypo-function	[24]

**Table 2:** Treatment outcome of clinical trials for PD-1/PD-L1 inhibitors combined with trastuzumab.

Drugs	Trial Registration Number	Effects	Adverse Reaction	Reference
Trastuzumab plus Pembrolizumab	NCT02129556	Objective response from 15% of PD-L1-positive cancer patients and 39% of patients with TIL counts over 5%; 25% of patients in the PD-L1-positive subgroup achieved disease control.	Grade 3–5 ADs related to treatment occurred in 17 (29%) and serious ADs occurred in 29 (50%) patients. Common severe ADs included dyspnoea, pneumonitis, pericardial effusion, and upper respiratory infection.	[21,56]
Trastuzumab plus Durvalumab	NCT02649686	Objective response was not observed; stable disease at week 6 in 4 of 14 PD-L1-negative cancer patients.	Diarrhea (13%), arthralgia (7%), paresthesia (7%), rash (20%).	[21,57]
Trastuzumab emtansine plus Atezolizumab	NCT02924883	Median PFS was 8.2 months for atezolizumab-treated patients, slightly longer than those assigned placebo.	Severe AEs could be observed in 43 of 132 (33%) atezolizumab-treated patients. Common grade 3 or worse AE was thrombocytopenia (13%).	[58]

**Table 3:** Related drugs of PD-1/PD-L1 inhibitors combined with molecularly targeted therapy.

Drugs	Target	Trial Registration Number	Effects	Adverse Reaction	Reference
Duvalizumab plus Olaparib	PD-L1, PARP	NCT02734004	24 of 30 (80%) patients achieved disease control at week 12 (primary efficacy endpoint). There were 15 patients (50%) achieving disease control at week 28. Median response duration was 9.2 months.	11 of 34 (32%) patients presented grade 3 or worse AEs; common AEs included neutropenia, pancreatitis, and anemia. Treatment for some patients stopped because of AEs.	[59]
Niraparib plus Pembrolizumab	PARP, PD-1	NCT02657889	Among the 57 female patients, 5 patients gave complete responses, 5 gave partial responses, 13 suffered stable disease, and 24 suffered progressive disease.	Common grade 3 or worse AEs associated with treatment included anemia (10.18%), thrombocytopenia (8.15%), and fatigue (4.7%). Eight immune-related patients (15%) presented AEs related to immune, and two patients (4%) presented grade 3 AEs.	[64]
Cabozantinib (XL184)	MET and VEGF receptor 2 (VEGFR2, RET, KIT, AXL, and FLT3)	/	Tumor pathology is changed, which weakens the proliferation of tumor and endothelial cells, and enhances apoptosis and inhibits tumor growth in a dose-dependent manner.	/	[65]
Bisindole-PBD (5b)	VEGF	/	Regulate the proliferation of BC cells by inhibiting angiogenesis.	/	[66]
Emodin	Nuclear receptor corepressor 2 (NCOR2)	/	Transcriptional regulators (NCOR2 and SerRS) are targeted for suppressing VEGFA transcription and tumor angiogenesis.	The pharmacokinetic properties are poor, i.e., the bioavailability, AUC, and Cmax are low, hence not proper for clinical treatment.	[67]
Salinomycin (SAL)	HIF-1α/VEGF mediated tumor angiogenesis	/	SAL interrupts HIF-1α/VEGF signaling for inhibiting angiogenesis and BC growth induced by VEGF and could be used as a promising antiangiogenic agent for BC treatment.	At higher doses, SAL may have some adverse effects.	[68]
Bevazhu monoclonal antibody	All known VEGF-A subtypes	/	Therapeutic activity for locally recurrent and metastatic BC.	The adoption of VEGF pathway targeting drugs to inhibit angiogenesis causes many classic side effects, represented by hypertension (and proteinuria).	[69]

**Table 4:** Related drugs of nanotechnology combined with immunotherapy.

Drugs	Effects	Reference
TiDMSN plus ICD and ICB	This improved the therapeutic effect of ICD strategy. TiDMSN is capable of significantly enhancing the treatment effect and hemolytic effect.	[85]
PS3D1@DMXAA plus ICB	12.5% of mice receiving PS3D1@DMXAA+anti-PD-1 treatment presented complete regression and did not suffer tumor burden for less than 50 days following the treatment.	[84]
